# Biomarkers Identification in the Microenvironment of Oral Squamous Cell Carcinoma: A Systematic Review of Proteomic Studies

**DOI:** 10.3390/ijms25168929

**Published:** 2024-08-16

**Authors:** Silvia Pomella, Ombretta Melaiu, Loredana Cifaldi, Roberto Bei, Marco Gargari, Vincenzo Campanella, Giovanni Barillari

**Affiliations:** Department of Clinical Sciences and Translational Medicine, University of Rome Tor Vergata, Via Montpellier, 00133 Rome, Italy; silvia.pomella@uniroma2.it (S.P.); ombretta.melaiu@uniroma2.it (O.M.); cifaldi@med.uniroma2.it (L.C.); bei@med.uniroma2.it (R.B.); marco.gargari@gmail.com (M.G.); vincenzo.campanella@uniroma2.it (V.C.)

**Keywords:** biomarkers, tumor microenvironment, oral squamous cell carcinoma, proteomic, stroma, CAFs

## Abstract

An important determinant for oral squamous cell carcinoma (OSCC) onset and outcome is the composition of the tumor microenvironment (TME). Thus, the study of the interactions occurring among cancer cells, immune cells, and cancer-associated fibroblasts within the TME could facilitate the understanding of the mechanisms underlying OSCC development and progression, as well as of its sensitivity or resistance to the therapy. In this context, it must be highlighted that the characterization of TME proteins is enabled by proteomic methodologies, particularly mass spectrometry (MS). Aiming to identify TME protein markers employable for diagnosing and prognosticating OSCC, we have retrieved a total of 119 articles spanning 2001 to 2023, of which 17 have passed the selection process, satisfying all its criteria. We have found a total of 570 proteins detected by MS-based proteomics in the TME of OSCC; among them, 542 are identified by a single study, while 28 are cited by two or more studies. These 28 proteins participate in extracellular matrix remodeling and/or energy metabolism. Here, we propose them as markers that could be used to characterize the TME of OSCC for diagnostic/prognostic purposes. Noteworthy, most of the 28 individuated proteins share one feature: being modulated by the hypoxia that is present in the proliferating OSCC mass.

## 1. Introduction

The tumor microenvironment (TME) is a complex system constituted by a continuously remodeling extracellular matrix (ECM) and heterogeneous cell types [1]. The latter include transformed cells and non-malignant cells such as cancer-associated fibroblasts (CAFs), tumor-associated macrophages (TAMs), T cells, natural killer (NK) cells, B cells, endothelial cells or pericytes of blood and lymphatic vessels, and mesenchymal stem cells (MSCs) [1]. Dysregulation of the crosstalk among the various components of the TME alters cell survival, growth, and/or differentiation, ultimately leading to tumor progression [2]. Indeed, because of an imbalance in the concentration of bioactive molecules that are released in the TME, cancer cells further dedifferentiate, acquiring a stem cell-like phenotype (cancer stem cells, CSCs) that renders them very metastatic and resistant to antitumor therapies [3,4]. Concerning the non-malignant cells present in the TME, despite the stability of their genome, they are highly plastic, and their phenotype can be reprogrammed following the interaction with cancer cells and other cells of the TME [5]. For example, CAFs, which are the most abundant non-malignant cells in the TME, continuously switch to different phenotypes, including the myofibroblast, the immune-regulatory, or the antigen-presenting one [6].

Thus, the study of the molecules expressed and released by TME cells could significantly contribute to understanding the outcome of a given tumor, possibly allowing the identification of crucial markers for its early detection and its timely treatment.

Nowadays, proteomic approaches have shed light on the pathways through which the TME is modulated by proteins synthesized by the different cell types that are present therein [7]. 

Regarding proteomic techniques, mass spectrometry (MS) has identified and characterized tumor biomarkers that are now used in routine diagnostics [8]. 

MS is based on the separation of ions according to their mass-to-charge ratio; it relies on several types of mass analyzers, including quadrupole, magnetic sector, radio frequency ion trap, time-of-flight (TOF), orbitrap, and ion cyclotron resonance [8]. To separate the analytes of interest from the matrix components and to improve the sensitivity and specificity of the detection, liquid chromatography coupled to tandem mass spectrometry (LC-MS/MS) is used, whose strength lies in its high analytical selectivity [9].

Certainly, MS is very likely to provide useful information about oral squamous cell carcinoma (OSCC), an aggressive and highly metastasizing malignancy that originates from the transformation of epithelial cells lining the oral cavity, most frequently in the palate, the floor of the mouth, and the tongue [10]. Nowadays, OSCC accounts for over 90% of oral cancers [10], and it is witnessing a global increase in annual new cases, prominently in Asia, followed by Western countries; this places OSCC among the top 10 most common human malignancies [11]. 

The onset of OSCC is influenced by host genotype, age, and gender, and it is favored by bacterial periodontitis and/or lifestyle-related factors including smoking, alcohol consumption, exposure to ultraviolet radiation, and the use of betel quid [12,13]. Infection of oral keratinocytes by human papilloma viruses (HPVs) is another risk factor for OSCC development, which occurs mostly in young individuals [14]. 

Treatment options for OSCC range from surgery and radiation therapy to chemotherapy or a combination thereof, being selected based on the severity of the disease [15,16]. However, these approaches have significant side effects [17]. Moreover, OSCC patients, after an initial response, often develop chemo- and/or radio-resistance [18]. Consequently, OSCC has a poor prognosis, and the survival rates of OSCC patients have seen minimal improvement over the last decades [19].

In this regard, one should consider that conventional antitumor therapies, such as chemotherapy and radiotherapy, are mainly directed against cancer cells [20]. In recent years, however, anticancer treatments have been individualized to be alternative or complementary to conventional ones. For instance, the discovery of immune checkpoints has allowed the boosting of antitumor immune responses [21]. More recently, stromal cells have been shown to play a critical role in tumor development, progression, and therapy resistance, so antitumor treatments that take stromal cells into account are becoming equally important [22].

In this context, the understanding of the proteins that drive OSCC progression is limited. Recently, however, two articles have reported that in OSCC tissues, the cancer cells, the CSCs, CAFs, MSCs, and the TAMs interact via the proteins they release, thereby establishing a niche that supports tumor progression [23,24]. It is quite likely that those and other TME proteins could work as biomarkers for diagnostic purposes (possibly allowing OSCC early detection) and/or as targets of novel therapeutic approaches (hopefully more effective than the ones adopted nowadays). 

Aimed at providing information that could be used to improve the specificity and sensitivity of the diagnostics of OSCC and, possibly, to streamline the therapeutic options directed against that tumor, this systematic review summarizes and integrates available data on the proteins that are found in the TME of OSCC, as identified through proteomic approaches. 

## 2. Materials and Methods

### 2.1. Systematic Review Protocol

The present study has relied on Preferred Reporting Items for Systematic Reviews and Meta-Analyses (PRISMA) guidelines [25]. The registration number is INPLASY2023120114. The DOI number is 10.37766/inplasy2023.12.0114.

### 2.2. Search Strategy and Eligibility Criteria

The PubMed/MEDLINE, Embase, Scopus, and Web of Science databases were used to perform a systematic search for articles published between 1 January 2001 and 31 December 2023, using the following search query: (tumor microenvironment) AND (oral squamous cell carcinoma) AND (proteomic). Inclusion criteria were articles published in the English language containing original data, reporting results of human patient samples, describing cases of OSCC, and applying proteomic-based techniques. Exclusion criteria were reviews, conference abstracts, letters, retracted articles, and in vitro or in vivo preclinical studies. Two reviewers (S.P. and O.M.) have performed an independent review of the abstract and full text of the retrieved articles. Articles meeting the inclusion criteria were selected for a comprehensive, final systematic review. The quality assessment for risk of bias was realized using the Joanna Briggs Institute Critical Appraisal tool.

### 2.3. Methodological Quality Assessment and Data Extraction

**Figure 1:**
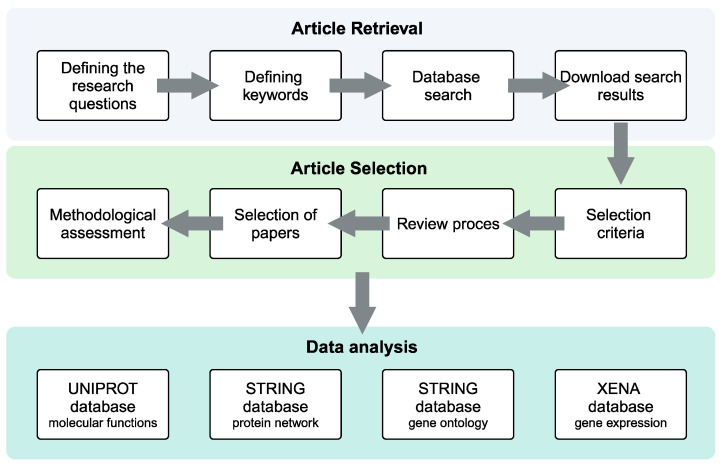
Schematic workflow of the systematic review process.

Two independent reviewers (S.P. and O.M.) have extracted the data and evaluated their quality. All the authors of the present review addressed potential disagreements through discussions. The screening of the articles, duplicate exclusion, and the reasons for exclusion were documented and recorded using Rayyan [26]. The following information was extracted: author(s), year of publication, sample characteristics and numbers, and techniques involved in proteomics. Potential protein biomarkers retrieved from the studies were characterized by collecting protein names, protein IDs, and molecular functions in the UniProt database (https://www.uniprot.org (accessed on 27 January 2024)) [27]. Protein association networks were retrieved and downloaded from the STRING database, in which known and predicted protein–protein interactions are annotated (http://string-db.org (accessed on 27 January 2024)) [28]. The following default settings were applied to the STRING network: network type (full STRING network), meaning of network edges (evidence), active interaction sources (text mining, experiments, databases, co-expression, neighborhood, gene fusion, and co-occurrence), and minimum required interaction score (medium confidence 0.400). Gene Ontology (GO) Enrichment Analysis of the identified proteins was applied to identify consistent correlations across the group of proteins and their classifications for “biological processes”, “molecular functions”, and “cellular components” using the default settings of the STRING database. The following network stats were obtained: number of nodes 28, number of edges 45, average node degree 3.21, average local clustering coefficient 0.583, expected number of edges 13, PPI enrichment *p*-value 4.39 × 10^−12^”. RNA count data and clinical information from The Cancer Genome Atlas (TCGA) for head and neck squamous cell carcinoma (HNSCC, PanCancer Atlas, Bethesda, MD, USA) and normal tissues were downloaded from the Xena database. The base of the tongue, oral tongue, oral cavity, buccal mucosa, hard palate, and floor of the mouth were chosen as the anatomic sites that defined the OSCC patient subset. A one-way ANOVA for multiple comparisons was applied to calculate the statistical significance. Only comparisons with a *p*-value ≤ 0.05 were considered significant. The data were analyzed and plotted as box plots using GraphPad Prism 8.0.2 (for Windows, GraphPad Software, Boston, MA, USA, www.graphpad.com) The schematic workflow of the systematic review process is reported in Figure 1.

## 3. Results

### 3.1. Systematic Review Analysis 

**Figure 2:**
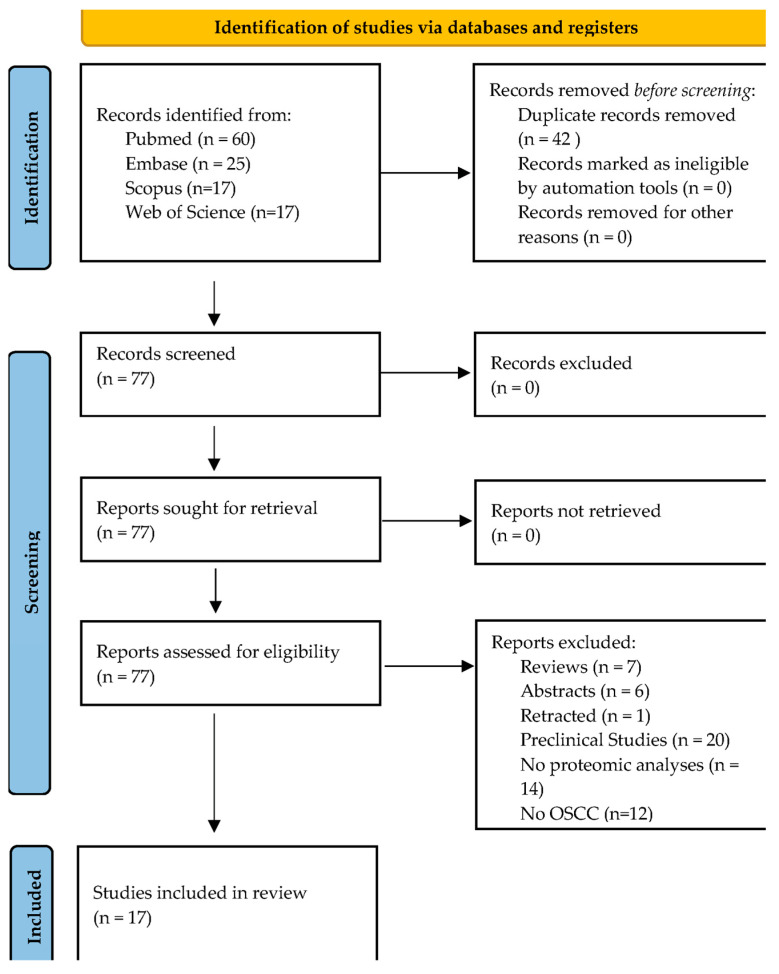
Representation of the PRISMA workflow (http://www.prisma-statement.org (accessed on 27 January 2024)) for the selection of articles.

**Figure 3:**
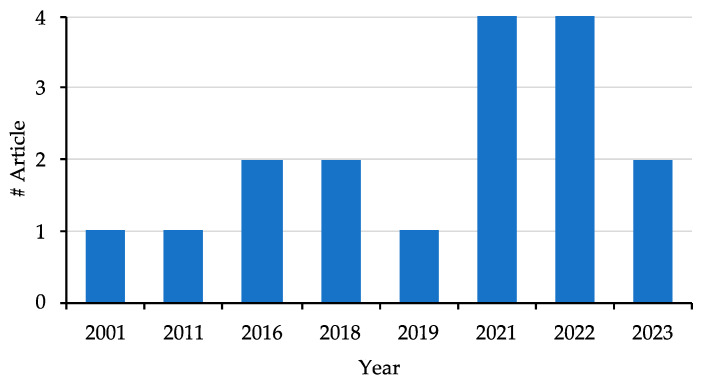
A bar graph depicting the number and year of publication of the selected articles.

The literature search strategy has resulted in 119 articles published between 2001 and 2023. The systematic search process is reported in Figure 2. Forty-two articles were removed because they were duplicates. Of the remaining 77 articles, only 17 were found to be fully compliant with the selection criteria (Figure 2 and Figure 3 and Table 1). Among the 60 excluded articles, 7 were reviews, 6 were conference abstracts, 1 was a retracted article, 20 were preclinical studies, 14 articles did not report data from any type of proteomics, and 12 articles did not focus on OSCC. Sixteen out of 17 studies were classified as having an overall low risk of bias; one study had an overall moderate risk of bias (Joanna Briggs Institute Critical Appraisal) (Supplementary Figure S1) [29]. Each study was independently evaluated and judged on the domain (D) of the tool by S.P. and O.M. The studies were rated as at low risk of bias (when the study reaches a score of more than 70%, “yes”), moderate (50–69%), or high (less than 49%).

Of the 17 selected articles, 8 have performed proteomics studies on the entire tumor mass. Specifically, six have focused on the proteome (i.e., the entire set of proteins expressed by the selected tissue), one has analyzed both the proteome and the phosphoproteome (i.e., the set of proteins that contain a phosphate group as a post-translational modification), and one has analyzed the secretome (i.e., proteins secreted into a conditioned medium, analyzed by proteomic techniques after culturing in vitro a fraction of the total tumor mass or a single cellular component of TME, capable of releasing multiple factors into the medium itself) of human OSCC specimens.

Concerning the other nine papers analyzed in this review, seven have focused exclusively on OSCC stroma, with CAFs and/or MSCs being the first matrices to be sorted. Of these seven papers, two have carried out studies on the secretome, one on both the secretome and the proteome, and four on the proteome. The remaining two papers have performed studies on the proteome or secretome by analyzing the tumor interstitial fluid (TIF). 

Of note, the blind search, systematically conducted without a priori hypotheses, did not reveal any study in the OSCC TME investigating the lysine acetyl and (redox)-based post-translational modification (redox PTM) proteomes, an aspect that deserves further investigation in future experimental studies.

The data we have retrieved from the 17 selected studies involve OSCC specimens from a total of 108 patients. In 13 of the 17 studies, the cohort of patients with OSCC was compared to a cohort of healthy individuals, with a total of 59 non-tumoral samples that were considered herein. Patients with tumor recurrence, oral metastasis of systemic tumors, a history of radiotherapy and chemotherapy, and other severe systemic diseases were excluded from this study. As shown in Table 1, all proteomics analyses were performed on samples collected from treatment-naïve patients, that is, patients who underwent surgical tumor resection prior to chemotherapy and/or radiotherapy. Only [30] includes a patient who, prior to surgery, received short-term chemotherapy and radiotherapy. 

Overall, the statistical methods used to analyze the proteomic data were consistent across the selected articles. In 8 of the 17 publications, a subset of the proteomic data found to be statistically significantly deregulated was validated with other technologies (i.e., IHC, IF, ELISA, and/or WB). In each case, validation of the omics results was achieved (Supplementary Table S1).

**Table 1:** Characteristics of the selected articles.

Refs	Cell Type	# Patient	# Control	Cohort of Patients	Sample Type	Technique
[31]	Tumor mass epithelium/stroma	2	4	Treatment-naïve patients	Proteome	Antibody microarray
[32]	Tumor mass	5	5	Treatment-naïve patients	Proteome	Imaging mass spectrometry associated with MALDI-TOF ultrafleXtreme
[33]	Tumor mass	15	/	Treatment-naïve patients	Proteome	Tandem mass tags LC-MS
[34]	Tumor mass center/adjacent epithelium	3	/	Treatment-naïve patients	Proteome	Dimethyl labeling associated LC-MS based on LTQ-Orbitrap Velos coupled with HPLC
[35]	Tumor mass	5	5	Treatment-naïve patients	Secretome	LC associated with nano-liquid MS
[30]	Tumor mass	15	5	All treatment-naïve patients, but one	Proteome/ Phosphoproteome	Tandem mass tags LC-MS
[36]	Tumor mass	14	14	Treatment-naïve patients	Proteome	LC-MS
[37]	Tumor mass	20	/	Treatment-naïve patients	Proteome	LC-MS
[38]	CAFs	4	4	Treatment-naïve patients	Secretome/ Proteome	Qexactive equipped with an EASY-Spray source tandem MS
[39]	CAFs	1	1	Treatment-naïve patients	Secretome	LC-MS
[40]	ECM	6	3	Treatment-naïve patients	Proteome	LC-MS
[41]	CAFs	1	1	Treatment-naïve patients	Secretome	MS based on LTQ-Orbitrap XL coupled to a nanoACQUITY Ultraperformance LC
[42]	CAFs	1	/	Treatment-naïve patients	Proteome	Phosphoproteomics by MS with Nano-LC
[43]	CAFs	3	3	Treatment-naïve patients	Proteome	Label-free LC-MS
[44]	MSCs	1	2	Treatment-naïve patients	Secretome	EASY-NLC 1000 Ultra-Performance LC system followed by MS in a Q Exactive TM Plus
[45]	TIF/NIF	10	10	Treatment-naïve patients	Proteome	In-gel digestion and GeLC-MS based on LTQ-Orbitrap Discovery coupled with HPLC
[46]	TIF/NIF	2	2	Treatment-naïve patients	Proteome	MS analysis on an LTQ-Orbitrap XL

Abbreviations: CAFs (cancer-associated fibroblasts); ECM (extracellular matrix); HPLC (High Performance Liquid Chromatography); LC (liquid chromatography); LC-MS (liquid chromatography associated with mass spectrometry); MALDI-TOF (Matrix-Assisted Laser Desorption Ionization—Time of Flight); MS (mass spectrometry); MSCs (mesenchymal stem cells); TIF (tumor interstitial fluid); NIF (normal interstitial fluid).

### 3.2. Proteomic Profiling of the Whole OSCC Mass

Early in 2001, Knezevi et al. studied the proteome features at various stages of OSCC progression. They found that the levels of ribosomal protein S6 kinase alpha-1, interferon alpha, glutamate receptor ionotropic, and retinoic acid receptor-alpha were increased in the stroma of invasive OSCC as compared to that of non-neoplastic oral epithelium. These results were validated through conventional techniques, such as Western blot and immunohistochemistry, and they pointed at retinoic acid receptor-alpha, that was found upregulated in the transition from in situ to invasive OSCC, as a biomarker of OSCC progression [31]. 

In 2016, Widlak et al. determined the molecular signatures that differ between OSCC and normal oral epithelium. Their analysis led to the identification of 108 upregulated and 26 downregulated peptides in OSCC compared to normal epithelium, with the overexpression of many molecules promoting cell survival or locomotion and the repression of some players of glucose metabolism [32]. 

In 2021, Routray et al. analyzed the proteomic profile of OSCCs at different grades of differentiation. Results indicated that ECM molecules produced by CAFs, such as periostin (POSTN), tenascin C (TNC), caveolin-1 (CAV1), and fascin [47,48,49], were expressed at high levels in moderately differentiated OSCC as compared to poorly differentiated OSCC. These findings elect the abovementioned molecules as biomarkers of OSCC differentiation grade [33]. 

Again in 2021, Alves et al. analyzed OSCC and non-neoplastic peritumoral epithelium, both tissues displaying low, intermediate, or high inflammatory infiltrates. The authors found 134 proteins that were differentially expressed in neoplastic and non-neoplastic tissues. Twenty of those proteins were related to the inflammatory response: of them, 11 were detected in all 3 types of inflammatory infiltrates, while 9 were expressed either in intermediate or high inflammatory infiltrates. Amidst the latter nine proteins, neutrophil defensin protein (DEFA) 1 and leukocyte elastase inhibitor were upregulated in OSCC when compared to uninvolved peritumoral epithelium. Conversely, histidine-rich glycoprotein and alpha-1-antichymotrypsin were downregulated in OSCC as compared to non-neoplastic epithelium [34]. In addition, Alves reported a 2-fold increase in M2 (pro-tumor) TAMs over M1 (antitumor) TAMs in OSCC as compared to non-neoplastic peritumoral epithelium [34]. Altogether, these results described a pro-inflammatory scenario in OSCC [34]. 

Still in 2021, Fraga et al. examined five OSCC secretomes, compared to five secretomes from non-neoplastic oral epithelium. Results from that analysis indicated that CAV1, protein disulfide-isomerase A3, phospholipase A-2-activating protein, calcium/calmodulin-dependent protein kinase type 2, and prostaglandin G/H synthase 2 were increased in OSCC secretomes as compared to their normal counterparts; in contrast, vitamin D-binding protein was downregulated in OSCC secretomes compared to those from non-neoplastic epithelium [35]. Because of the decrease in vitamin D-binding protein, vitamin D accumulated in OSCC, thereby triggering prostaglandin E production by TAMs and impairing the antitumor responses of T cells [35]. 

In 2022, Kaneko and colleagues analyzed HPV-positive, recurrent, and non-recurrent OSCCs. In recurrent OSCCs, the protein levels of DEFA3, matrix metalloproteinase (MMP)-8, MMP-9, neutrophil elastase, the alpha chain of fibrinogen (FGA), and the gamma chain of fibrinogen (FGG) were increased. Conversely, T-cell surface antigens such as CD2, CD3 gamma chain, CD6, and the alpha chain of CD8 were decreased; this suggested that OSCC recurrence is linked to impaired T-cell function [30]. Again in agreement with a dysfunction of T cells, the phosphoproteomic analysis revealed that recurrent OSCCs display a reduced activation of CD8, CD3, lymphocyte-specific protein tyrosine kinase, and the hematopoietic signal transducer VAV1. In addition, the phosphoproteomic analysis showed that recurrent OSCCs display active (phosphorylated) FGG, Signal Transducer and Activator of Transcription (STAT)5A/B, and Sodium-dependent Imino Transporter 1. Finally, Kaneko et al. reported that recurrent OSCC exhibits a pro-fibrotic and immunity repressing TME, characterized by a high number of ECM-producing CAFs and immunosuppressive MSCs [30]. 

Also in 2022, Mumtaz and colleagues examined protein expression profiles in tumor and adjacent normal tissues from 14 patients with OSCC. In addition, they performed secretome profiling of nine OSCC cell lines and bioinformatics analysis using publicly available datasets [36]. The authors found 205 highly upregulated proteins in OSCCs compared to non-neoplastic tissues. The majority of those proteins were involved in biological processes, including spliceosomal complex assembly, protein localization in the endosome, aminoacylation of tRNA for protein translation, immunity, and protein biosynthesis. Twenty-five proteins emerged from the integration of proteomic data with those obtained from the analysis of the secretome and of the publicly available databases. Amidst those proteins, THBS2, LGALS3BP, and DNAJB11 are of particular interest since previous studies designated them as potential salivary markers for OSCC diagnosis [50,51].

Recently, Miranda-Galvis and colleagues characterized the TME of 20 OSCCs (9 HPV-positive and 11 HPV-negative). The authors identified 39 proteins that were differentially expressed depending on the presence or absence of HPV. In particular, GO analysis revealed that RNA processing was one of the pathways activated in HPV-positive OSCCs. As for HPV-negative OSCCs, the mediators of immune responses were upregulated. Independently from HPV infection, the S100-A8 protein was overexpressed in OSCCs, as compared to normal controls, in a fashion negatively correlating with patients’ disease-free survival and overall survival. Consistent with the impact that S100-A8 has on the immune response and on inflammatory processes [52], the overexpression of S100-A8 altered the composition of the immune cell infiltrate, lowering the number of M1 macrophages and dendritic cells [37]. Altogether, these findings pointed to S100-A8 as being both a prognostic marker and a therapeutic target for OSCCs. 

### 3.3. Proteomic Profiling of the OSSC Stromal Microenvironment

Lately, several research groups have used MS-based proteomic assays to characterize the proteins that are present in OSCC stroma, especially those specifically synthesized by CAFs.

In this context, Principe et al. analyzed the whole cell lysate, conditioned media, and exosomes isolated from primary CAFs and adjacent fibroblasts of OSCC patients by proteomics and secretomics, respectively. They identified 944 proteins that were commonly expressed in all three fractions. Amidst the secreted proteins that were also enriched in CAF, of particular interest was the microfibril-associated protein 5, which promotes the growth and migration of OSCC cells due to its ability to activate both the mitogen-activated protein kinase (MAPK) and the AKT serine/threonine kinase 1 (AKT) pathways [38]. 

Consistently, Bagordakis et al. identified a series of proteins related to ECM organization, ECM degradation, and collagen metabolism whose levels were augmented in the secretome of CAFs as compared to that of normal oral fibroblasts. Among the most upregulated proteins, fibronectin type III-containing domain 1, serpin peptidase inhibitor type 1, and stanniocalcin 2 were suggested as potential new therapeutic targets. In addition, Bagordakis et al. showed that the presence of CAFs in the tumor stroma negatively correlates with the disease-free survival of OSCC patients [39]. 

In this regard, He et al. deprived the ECM of OSCC and normal oral mucosa from cells and assessed the quality and quantity of ECM proteins. The authors identified 26 proteins that were specifically present in the ECM of OSCCs. Among those proteins, 14 were typical of late-stage OSCCs. These results indicated that ECM composition continuously changed during OSCC progression. Such changes affected events linked to carcinogenesis, such as cell differentiation, apoptosis, metastasis, and new vessel formation [40]. 

Álvarez-Teijeiro et al. reported that cytokines secreted by CAFs (but not by normal fibroblasts) in SCC cells activated the receptors for epidermal growth factor, insulin-like growth factor, and platelet-derived growth factor; this promoted the survival and growth of SCC cells and their dedifferentiation into CSCs, which are key to tumor initiation, progression, recurrence, metastasis, and resistance to therapy. Of note, the pharmacological inhibition of the abovementioned receptors effectively blocked the establishment of the CSC phenotype, thus unveiling those receptors as novel targets of anti-OSCC therapies [41]. 

Prieto-Fernandez et al. focused on the paracrine communications that occur between cancer cells and stromal cells. Specifically, the authors reported that molecules released by HNSCC cells promoted in CAFs a rapid activation of a variety of signaling pathways. The latter included MAPK kinase 4, MAPK kinase 7, AKT, Raf-1 serine/threonine kinase (RAF), and B-Raf serine/threonine kinase (BRAF). This resulted in an increase in the expression of the ECM-degrading MMP-1, MMP2, MMP-8, MMP-9, and MMP-13, hence strengthening the invasiveness of SCC cells. These events were blocked by sorafenib, an inhibitor of RAF/BRAF that emerged as a promising candidate to counter head and neck SCCs and OSCC [42]. 

Based on the well-established alteration of the energy metabolism that is typical of cancer cells, Xiao et al. investigated the molecular differences existing between CAFs and normal fibroblasts in mitochondria. Among the many differentially expressed mitochondrial proteins, the authors focused on TNF-receptor-associated protein-1 (TRAP1), a member of the HSP90 family that regulates the switch between mitochondrial respiration and aerobic glycolysis. The authors found that, when it was overexpressed in OSCC-associated CAFs, TRAP1 reduced oxidative phosphorylation and promoted OSCC growth and progression [43]. 

Additional proteomic studies examined cellular components of the TME other than CAFs. In that context, Li et al. obtained exosomes from the MSCs of healthy oral mucosa and from OSCC-associated MSCs and showed increased levels of MMP-1 in the exosomes of the latter, which contributed to the pro-tumor activity of these cells [44]. Further study analyzed the OSCC interstitial fluid, as this specimen is enriched in a variety of cancer-related secreted molecules that could possibly be employed as diagnostic/prognostic markers. Using this type of matrix, Hsu et al. identified six pathways that were differentially expressed in TIF as compared to the interstitial fluids of non-tumoral tissues (NIF). Amidst those pathways, that associated with aminoacyl-tRNA biosynthesis was found to be the most dysregulated, suggesting its possible involvement in oral carcinogenesis. Moreover, the authors found nidogen-1 (NID1) among the proteins that were present at high levels in TIF but not in NIF. NID1 is an ECM molecule that, because of its binding to laminin and collagen IV, mediates the assembly of the basement membrane. Validation studies confirmed that NID1 salivary levels were significantly higher in OSCC patients than in healthy individuals. Of note, the authors had also observed that the increase in NID1 levels paralleled OSCC progression and was correlated with a poor prognosis [45].

Still concerning the TIF of OSCC, Hardt et al. combined the use of micro-dialysis with MS-based proteomics, which identified MMP-8 and MMP-9 among the molecules that were more abundant in TIF than in NIF [46].

### 3.4. TME Protein–Protein Interaction and Association Network

Collectively, the 17 studies we selected for the present review identified 570 proteins that were differentially expressed in OSCC as compared to normal oral mucosa. Despite some studies that validated a few of these proteins through other methods, diagnostic tests and correlations with clinical outcomes were not significant for many of them.

Among the identified proteins, 28 were cited in at least 2 different studies (Supplementary Table S2). Their molecular functions are reported in Table 2. 

Considering their function, the 28 proteins that we have identified can be divided into two groups: the mediators of cell–ECM adhesive interactions and the mediators of glycolysis.

#### 3.4.1. Molecules Mediating Cell Adhesive Interactions with the ECM

In this regard, it has to be highlighted that the interactions occurring between the cells and the ECM are profoundly altered in cancer tissues as compared to their non-neoplastic counterparts [53]. Such changes play a role in the dysregulation of cell differentiation, cell survival, cell growth, and cell migration that is typical of cancer cells [53]. Accordingly, we found that the expression level of the following molecules differs in OSCCs and normal oral mucosas:

(1) Calreticulin (CALR) is an endoplasmic reticulum protein that supports calcium-dependent processes, such as integrin-mediated cell adhesion to the ECM [54]. CALR expression has been found to be reduced in CAFs compared to normal fibroblasts [38,41].
(2) Chondroitin sulfate proteoglycan-4 (CSPG4), which consists of a transmembrane glycoprotein combined with a chondroitin sulfate proteoglycan, is expressed on the filopodia of migrating tumor cells, where it associates with the ECM-binding β1 integrins to favor tumor cell locomotion [55]. CSPG4 has been reported to be downregulated in CAFs and upregulated in TIFs when compared to their normal counterparts [41,45].
(3) (DEFA3), a small peptide that, in addition to neutralizing bacterial lipopolysaccharides [56], favors microbial adhesion and survival [57]. This could suggest similar effects exerted by DEFA3 on tumor cells. The levels of DEFA3 are increased in OSCCs [30,34].
(4) EGF-containing fibulin-like extracellular matrix protein 1 (EFEMP1) is a member of the fibulin family of ECM proteins that promotes cancer cell growth and invasion [58]. EFEMP1 has been reported to be diminished in the ECM of OSCCS and overexpressed in CAFs [40,41].
(5) Fibulin-2 (FBLN2), another fibulin family member, is highly expressed by normal epithelial cells as compared to carcinoma cells and, upon binding to the β1 integrins, inactivates both the MAPK and the AKT pathways, thereby inhibiting cell proliferation, migration, and invasion [59]. FBLN2 levels have been found to be reduced in the ECM of OSCCs and increased in CAFs [40,41].
(6) FGA represents the alpha chain of fibrinogen. The latter is a plasma protein whose cleavage to fibrin monomers is key to physiologic hemostasis [60], while in a tumor setting, it drives the formation of a fibrin matrix supporting cancer cell growth and metastasis [61]. FGA has been found to be downregulated in the ECM and upregulated in the mass of OSCCs [30,40].
(7) FGG is the gamma chain of fibrinogen. Analogously to FGA, FGG is also differentially expressed in the ECM and in the cellular mass of OSCC [30,40].
(8) Heterochromatin protein 1 binding protein 3 (HP1BP3), a ubiquitously expressed nuclear protein belonging to the H1 histone family, plays an important role in cell growth and viability [62,63]. In cancer tissues, HP1BP3 is involved in the adhesion of malignant cells to collagen [64]. However, studies evaluating HP1BP3 expression levels in OSCCs produced conflicting results [36,37]. This is likely to depend on the disparate genetic, epigenetic, and metabolic alterations observed in OSCC tissues obtained from different patients [36,37].
(9) Laminin γ2 subunit (LAMC2) is a glycoprotein that is one of the major non-collagenous components of basement membranes [65]. LAMC2 has been reported to be upregulated in both the TIF and the mass of OSCC [36,45].
(10) Latent transforming growth factor beta-binding protein 2 (LTBP2) is an ECM glycoprotein that impacts tumorigenesis because of its capability of regulating TGF-β activity and ECM remodeling [66,67]. LTBP2 has been found to be overexpressed in the mass and CAFs of OSCC [36,39].
(11) Lumican (LUM) is an ECM protein that maintains tissue integrity by regulating collagen fibrillogenesis [68]. LUM levels have been shown to be reduced in the CAFs of OSCC [38,41].
(12) The ECM-degrading MMP-1 is overexpressed by MSCs and downregulated in CAFs [38,42,44].
(13) The ECM-degrading MMP-3, about whose expression by CAFs conflicting data are reported [39,41].
(14) The ECM-degrading MMP-8 is found to increase OSCC mass, CAFs, and TIF [30,42,46].
(15) The ECM-degrading MMP-9 levels augment in the CAFs, TIF, and whole mass of OSCC [30,42,46].
(16) POSTN is a modulator of ECM assembly that is crucial to tissue development or repair [69] and that has been reported to be upregulated in both the TIF and the mass of OSCC [33,45].
(17) Transforming growth factor beta-induced (TGFBI) protein is an ECM component involved in cell adhesion, migration, and tissue organization [70]. TGFBI expression has been described as downregulated in the MSCs and upregulated in the CAFs of OSCC [39,44].
(18) TNC is an ECM protein that is involved in tissue development and repair [71] and that has been found to be overexpressed in the mass and TIF of OSCCs [33,36,45].

#### 3.4.2. Mediators of Glycolysis

Concerning the production of energy by cancer cells, it has to be reminded that the latter are frequently characterized by an impairment in mitochondrial function, leading to a reduction in oxidative phosphorylation and an increase in aerobic glycolysis [72]. In this context, our analysis revealed that, as compared to normal oral mucosas, OSCCs display abnormalities in the following molecules:

(1) Aldolase A (ALDOA) is a glycolytic enzyme that is highly prevalent within tumor cells. Its expression has been described as being reduced in MSCs and increased in CAFs [43,44].
(2) Carbamoyl-phosphate synthetase 2 (CAD) drives the expression of glycolytic enzymes [73]. CAD is upregulated in the OSCC mass [36,37].
(3) CAV1 is a component of cell membrane caveolae that is upregulated and/or highly phosphorylated in tumor tissues, thereby increasing glycolysis, reducing oxidative phosphorylation, and promoting cancer cell invasion and cancer metastases [72]. CAV1 has been shown to be upregulated in the OSCC mass [33,35].
(4) Ectonucleotide pyrophosphatase/phosphodiesterase family member 2 (ENPP2) is a target of the Kirsten rat sarcoma viral oncogene homologue (KRAS), a transcriptional activator of key players in glucose metabolism [74]. A previous study reported that ENPP2 expression is upregulated in tumor cells following β4 integrins binding to ECM molecules that, in turn, promote cancer cell invasion [75]. These findings provide a link between cell–ECM adhesive interactions and glycolysis. Expression levels of ENPP2 have been reported to be reduced in CAFs [39,41].
(5) Insulin-like growth factor 2 receptor (IGF2R) is a mediator of insulin and IGF-2 effects on glucose metabolism, cellular growth, and differentiation [76]. IGF2R is overexpressed in the OSCC mass and downregulated in the MSCs [36,44].
(6) Pyruvate kinase M (PKM) is an enzyme that catalyzes the final step of glycolysis by converting phosphoenolpyruvate to pyruvate, leading to the origination of ATP, a crucial source of energy for the cells [77]. PKM is overexpressed in CAFs [44], while its phosphorylated form (Y175) is downregulated in the OSCC mass [30,43].
(7) RNA binding motif protein 39 (RBM39) is a transcriptional coactivator whose expression increases in parallel with extracellular glucose levels, resulting in enhanced glucose utilization that supports cell growth [78]. RBM39 is upregulated in both OSCC mass and TIF [36,45].
(8) Splicing factor 3b subunit 3 (SF3B3) is a small nuclear ribonucleoprotein that alters tumor glycolysis in that it augments glucose consumption, lactate release, and extracellular acidification [79]. SF3B3 levels are increased in the OSCC mass [36,37].
(9) Voltage-Dependent Anion Channel 1 (VDAC1) is a mitochondrial protein that supports glycolysis by allowing the cytosol–mitochondria trafficking of metabolites [80]. Some studies have reported that VDAC1 expression is upregulated in CAFs, while others found it downregulated [38,43].

Finally, our search individuated that differences exist between OSCC and non-neoplastic oral tissue about the expression level of STAT5A [30,31]. The latter is a transcriptional activator of genes coding for proteins that are involved in both cell adhesion and glycolysis [81]. Specifically, STAT5A activation is followed by the transdifferentiation of epithelial cells, which first acquire a mesenchymal-like phenotype and then CSC features, including the production of growth-supportive and pro-invasive molecules, such as fibronectin, that are typical of the provisional ECM [82]. At the same time, the STAT5A mitochondrial fraction interacts with the pyruvate dehydrogenase complex, thereby promoting aerobic glycolysis while reducing oxidative phosphorylation [83]. 

To study the expression levels of the protein-coding genes, we selected 321 patients reported in the TCGA who were affected by HNSCC (see Materials and Methods). RNA-seq data for only 26 out of the 28 queried genes were available in the Xena database, and they were retrieved. Tumor samples were compared to matched normal oral tissues (*n* = 32; Supplementary Figure S2). 

Eleven out of 26 investigated genes were overexpressed in a statistically significant fashion in the OSCC cohort as compared to their normal counterparts. Such genes included *CAV1*, *CSPG4*, *LAMC2*, *LUM*, *MMP-1*, *MMP-3*, *MMP-8*, *MMP-9*, *POSTN*, *TGFBI*, and *TNC* (Supplementary Figure S2). As for *CAV1*, *LAMC2*, *MMP-8*, *MMP-9*, *POSTN*, and *TNC,* their mRNA levels agreed with the protein ones. Concerning other investigated genes, the mRNA levels of *CAD*, *CALR*, *FGA*, *FGG*, *IGF2R,* and *LTBP2* were increased, while those of *ALDOA*, *EFEMP1*, *ENPP2*, and *VDAC1* were decreased in OSCCs compared to normal tissues (Supplementary Figure S2). The mRNA expression of *CAD*, *LTBP2*, and *ENNP2* agreed with that of the protein. Regarding *FBLN2*, *HP1BP3*, *RBM39*, *SF3B3,* and *STAT5*, their mRNA levels did not differ in OSCCs as compared to non-neoplastic tissues.

**Figure 4:**
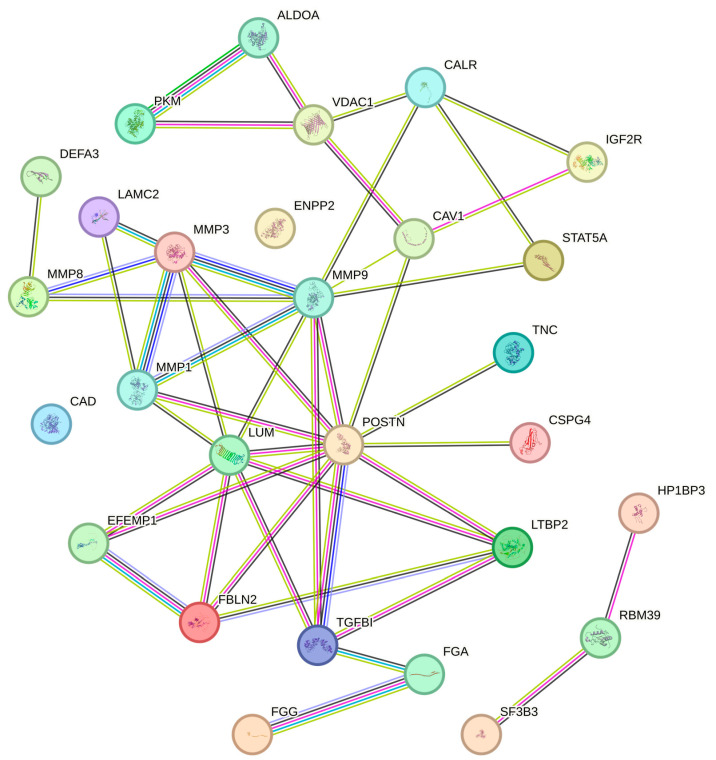
STRING association network of the 28 selected proteins. Network nodes represent proteins with their 3D-known structures. Blue line: known interaction from curated databases. Pink line: known interaction experimentally determined. Green line: predicted interactions from gene neighborhood. Red line: predicted interactions from gene fusions. Dark blue line: predicted interactions from gene co-occurrence. Yellow line: interactions by text mining. Black line: interactions by co-expression. Purple line: interaction by protein homology.

Network analysis, retrieved from the STRING database, revealed a protein–protein interaction and association network of 26 of the 28 identified proteins (Figure 4). 

Furthermore, GO analysis within the STRING database has revealed “ECM organization” and “collagen catabolic process” among the “biological processes” that are significantly enriched in OSCCs (Table 3).

Analogously, STRING pathway analysis for significative “molecular functions” associated with the selected proteins has identified “ECM constituent” and “metalloendopeptidase activity” as the most enriched ones (Table 4). 

In agreement with these findings, “ECM” and “collagen-containing ECM” have been found to be the most significantly enriched “cellular components” in OSCCs (Table 5).

## 4. Discussion

In this systematic review, we selected and summarized studies focused on the identification of deregulated proteins in the TME of OSCC through MS-based proteomics, an emerging diagnostic tool for several disease areas [84]. Results from STRING and GO analyses have highlighted the importance that TME proteins involved in cell–ECM interactions and glycolysis have in the development and progression of OSCC.

The proteins found dysregulated in the TME of OSCC from at least two different studies are: ALDOA, CAD, CALR, CAV1, CSPG4, DEFA3, EFEMP1, ENPP2, FBLN2, FGA, FGG, HP1BP3, IGF2R, LAMC2, LTBP2, LUM, MMP-1, MMP-3, MMP-8, MMP-9, PKM, POSTN, RBM39, SF3B3, STAT5A, TGFBI, TNC, and VDAC1.

The link that these proteins have with OSCC pathogenesis has also been suggested by studies based on techniques other than proteomics. Specifically, results from immunohistochemical analyses indicate that STAT5A is activated [85] and CALR [86], CAV1 [87], LUM [88], PKM [89], POSTN [90], and TGFBI [91] proteins are overexpressed in OSCC tissues as compared to their normal counterparts, which is associated with the poor survival of OSCC patients. Analogous results have been obtained about ALDOA [92] and ENPP2 [93] in studies based on RNA analysis. Similarly, enzyme-linked immunosorbent assays have shown an increase in MMP-1 and MMP-3 protein levels in specimens from OSCC patients [94]. In addition, MMP-1, MMP-3, MMP-8, and MMP-9 levels have been reported to increase in both OSCC tissue and the saliva of OSCC patients [95]. The expression patterns of MMPs were analyzed by immunohistochemistry, protein chip array, and RT-qPCR [95]. Moreover, biochemical analyses have revealed augmented levels of fibrinogen degradation products in the serum of OSCC patients as compared to healthy controls [94]. As for TNC, its expression has been shown to be upregulated in preclinical models of OSCC [96]. In contrast, a reduction in VDAC1 expression has been observed in OSCC and in dysplastic oral mucosa as compared to oral hyperplasia [97]. Regarding SF3B3, it has been indicated as one of the main promoters of leukoplakia transformation into OSCC [98]. Another study has shown a single nucleotide polymorphism with amino acid substitution from serine to asparagine at codon 811 (S811N) in exon 18 of this gene in OSCC patients [99]. As for LTBP2, it affects OSCC cell proliferation and invasion via the activation of AKT signaling [66]. Finally, concerning CAD, CSPG4, DEFA3, EFEMP1, FBLN2, HP1BP3, IGF2R, LAMC2, or RBM39, their contribution to OSCC has not been clarified in full.

Many of the results obtained in OSCC specimens agree with findings related to other tumor types. In fact, the glycolytic enzyme ALDOA has been shown to foster the growth and metastasis of tumors such as liver or lung carcinomas, osteosarcoma, and others [100]. Moreover, ALDOA levels in tumor tissues negatively correlate with the number of infiltrating immune cells, including macrophages, CD4^+^ T cells, and CD8^+^ T cells [100]. CAD has been reported to be enriched in several cancers (e.g., liver, breast, and colon carcinoma), where its expression correlates with poor clinical outcomes [101]. In HNSCCs other than OSCC, the expression of CAD is positively associated with that of glycolytic genes [73], and CAD antagonists inhibit cancer cell survival [102]. 

ENPP2 is involved in the clinical progression of breast carcinoma [103], and CALR exerts pro-tumor activities that are consistent with its effects on gene expression, cell proliferation, and immune response [104]. On its part, DEFA3 is overexpressed in both benign and malignant tumors of the salivary glands [105]. Regarding the products of fibrinogen degradation, FGA influences tumor cell proliferation and survival [106], and high FGG levels are found in late-stage liver carcinoma, which is associated with lymph node invasion, tumor relapse, and patients’ short overall survival [107]. HP1BP3 is overexpressed in thyroid or prostate carcinoma and in a few glioma types [108]. Moreover, HP1BP3 promotes esophageal carcinoma metastasis by upregulating miR-23a [109].

Regarding IGF2R, it has been found overexpressed in carcinomas of the larynx, uterine cervix, or bladder, as well as in melanoma, as compared to the non-cancerous peritumoral tissue [110,111,112,113].

LAMC2 expression is significantly upregulated in gastric carcinoma [114], pancreatic ductal adenocarcinoma [115], and in various HNSCC types [116,117,118,119].

LTBP2 is overexpressed in carcinomas of the uterine cervix [120], stomach [121], colon [122], pancreas [123], or HNSCCs [124], where the intensity of LTBP2 expression is inversely related to patients’ survival. In prostate carcinoma, LTBP2 protein increases in parallel with CD4+ T-cell infiltration and immune checkpoint blockade [125]. 

The levels of POSTN [69] and TNC [126] increase in tumors such as breast, lung, and pancreas carcinomas, where they interact with other ECM molecules to promote cancer cell survival, invasion, and metastatic spreading. 

Increasing evidence indicates that RBM39 influences the growth of a range of malignancies, including cholangiocarcinoma, rectum adenocarcinoma, kidney clear cell carcinoma, kidney papillary cell carcinoma, lung SCC, and HNSCCs [127]. Noteworthy, RBM39 expression reveals different prognostic outcomes for different tumors. Specifically, in hepatocellular carcinoma, adenoid cystic carcinoma, pheochromocytoma, and various sarcomas, high levels of RBM39 are associated with patients’ poor overall survival; in contrast, the overexpression of RBM39 is linked to the prolonged overall survival of women affected by uterine carcinoma [127].

As for SF3B3, its overexpression is associated with breast carcinoma’s late stage of progression and the poor survival of the patients [128]. 

STAT5A signaling [129], CSPG4 [130], MMP-1 [131], MMP-3 [132], MMP-8 [133], MMP-9 [134], and LUM [135] are all involved in cancer cell migration, invasion, and metastasis, while PKM supports the growth and survival of cancer cells by favoring aerobic glycolysis [136]. 

Concerning CAV1, EFEMP1, FBLN2, TGFBI, or VDAC1, their actions are highly context-dependent and can vary across different cancer types and stages. Indeed, cancer metastasis and patients’ poor prognosis have been associated with CAV1 downregulation in ovarian, breast, and lung carcinomas and with CAV1 upregulation in bladder or nasopharyngeal carcinoma [137]. Similarly, the downregulation of FBLN2 leads to the migration and invasion of breast cancer cells [138], while the binding of FBLN2 to the β chain of the integrin receptors promotes the growth of colorectal carcinoma [139]. In addition, increased TGFBI mRNA levels correlate to a better prognosis in non-small cell lung cancer patients and a poorer prognosis in esophageal SCC patients [140]. Moreover, VDAC1 expression is upregulated in breast carcinoma [141] and downregulated in endometrial carcinoma [142]. Finally, EFEMP1 acts as a tumor suppressor in prostate carcinoma [143], while most members of the fibulin family are crucial to tumorigenesis [144]. 

Noteworthy, 20 out of the 28 proteins identified in our review as key for OSCC progression are known to be differentially expressed in hypoxic tissues. Specifically, the levels of CALR, CSPG4, MMP-1, MMP-3, MMP-8, MMP-9, POSTN, TGFBI, TNC, ALDOA, IGF2R, PKM, FGA, and VDAC1 increase during hypoxia [145,146,147,148,149,150,151,152,153,154,155,156,157,158]. Again, the expression of CAD and CAV1 is activated by hypoxia-inducible transcription factor (HIF)-1 [159,160], while hypoxia sparks STAT5 and increases its DNA-binding activity in epithelial cells [161]. Moreover, EFEMP1 synthesis parallels hypoxia-induced angiogenesis [162]. In addition, hypoxia stabilizes the RBM39 protein, preventing its degradation by the cellular proteasome [163]. In contrast, LUM expression is repressed during hypoxia [164]. Altogether, these findings strengthen the clinical significance of our data. Indeed, hypoxia accompanying the rapid growth of OSCC mass exacerbates the aggressiveness of this tumor and worsens the patients’ prognosis [165,166]. We have not found any information about hypoxia effects on DEFA3, FBLN2, FGG, HP1BP3, LAMC2, LTBP2, ENPP2, or SF3B3; further studies will shed light on this topic.

Of note, the expression of 12 out of the 28 proteins identified in our systematic analysis is influenced by tobacco smoking or alcohol ingestion, which are the major risk factors for OSCC development [167,168]. Specifically, among the proteins differentially expressed in the oral mucosa, saliva, or lung of smokers and non-smokers were CALR [169,170,171], CAV1 [172], MMP-1 [173,174], MMP-8 [175], ALDOA [176], POSTN [177], PKM [178], and TNC [179].

Alcohol consumption increases the protein expression of ALDOA [180], FGA [181], FGG [182], LAMC2 [183], MMP-9 [184], and TGFBI [185]. In contrast, alcohol administration results in a decrease in CAV1 [186], while the expression of CALR [187], MMP-1 [188,189], and 3 [190] is not influenced by ethanol intake. 

These findings suggest monitoring the development and clinical progression of OSCCs by selecting diagnostic markers according to the patient’s voluptuary habits.

Our analysis, however, has several limitations.

As first, many of the studies included in our systematic review utilized a very small number of samples, and this certainly challenged the drawing of robust and reliable conclusions. 

In addition, it must be highlighted that identifying the diagnostic–prognostic markers was made difficult by the unlikeliness of OSCC patients and tumor specimens herein considered. 

Moreover, the interpretation of proteomic data may not have been uniform because of the different techniques and sample sizes that were used in the MS-based analyses considered here. 

Furthermore, one should consider that while the genome is relatively static, the proteome is extremely dynamic, with splice variants, glycosylations, phosphorylations, acetylations, methylations, ubiquitinations, and farnesylations [191]. Given that, the proteome contains >1000 times more cellular information than the genome, with >100,000 transcripts and potentially millions of protein variants [192]. To date, proteomic studies of OSCC lack such detailed information that would surely provide a powerful tool for understanding the biology of this tumor and for identifying potential biomarkers and therapeutic targets. 

Finally, other reliable diagnostic/prognostic markers for OSCC could be identified by techniques other than those based on proteomics, on which the present review has specifically focused.

Nonetheless, despite all these pitfalls, 108 different OSCCs were examined via our systematic search, allowing the identification of proteins that were consensually dysregulated despite the heterogeneity of the patients and tumor samples. Moreover, in 8 of the 17 selected publications, validation of a set of proteins found to be dysregulated by proteomic analyses was reported. In all of them, validation of the omics results was achieved. Of note, some of the proteins that proteomic analyses found to be dysregulated had been validated with techniques other than proteomics.

To sum up, our analysis has identified a group of 28 proteins that several researchers have reported as dysregulated in OSCCs compared to non-neoplastic oral mucosa. Overall, our results recommend further validating each of these proteins in larger prospective cohorts, also via the use of methodologies (e.g., ELISA, microarrays, or flow cytometry) that are routinely employed in clinical laboratories. The proteins that will be confirmed as differentially expressed in biological samples from OSCC patients could possibly be used in the diagnosis of this tumor.

Definitely, the data described in this review should be interpreted with caution, and additional research with a larger number of OSCC samples is needed to validate the observations herein reported. Thus, our systematic review should be considered a source of initial, preliminary insights into a field where data are still emerging. Altogether, the findings described here provide topics for future investigations, highlighting molecules that deserve to be further exploited as potential biomarkers for OSCC.

In this regard, there are specific experimental models [193] that could confirm and delineate a role in oral carcinogenesis for the proteins herein identified, as well as their application in OSCC diagnostics and treatment.

Integrating proteomics with OSCC preclinical models could help in both understanding the reliability of the molecules identified in this systematic review and generating molecular profiles that are eventually employable to guide personalized diagnostics and treatments.

Indeed, it is well established that an accurate prediction of the patients’ clinical outcome is achieved not only by examining many patients but also by evaluating the features of each patient’s tumor [194]. This can be accomplished by using two main approaches: (1) analyzing the OMICS big data, including but not limited to proteomics, that are related to tumor clinical course [194,195] and (2) developing customized platforms to test the biological behavior and the drug sensitivity of patient-derived samples. Such platforms are based on preclinical systems, which, as far as OSCC is concerned, include in vitro, ex vivo, and animal models [196,197,198]. While monolayer cell cultures are still the commonest approach for the initial evaluation of new therapeutics [199,200], three-dimensional (3D) organoid cultures of head and neck SCC better represent the tumor architecture and microenvironment and more reliably predict drug efficacy in patients [201]. Thus, MS proteomics of 3D models of OSCC could characterize OSCC heterogeneities among different patients, correlating molecular and cellular differences with the patient’s response to chemotherapy. This would greatly help to define more effective, less toxic personalized anticancer therapies.

## 5. Conclusions and Future Perspective

Most of the structural and signaling molecules of tumor cells and most of the targets of anticancer drugs are proteins in nature. Assessing the level and quality of proteins in tumor tissues is essential to obtaining information on cancer biology, as they are the reflection of tumor-associated genetic and epigenetic alterations [202,203]. As for OSCC, to date, the IHC has measured its protein biomarkers for a limited set of proteins, including Ki-67, p53, CK 17, CK 13, laminin-5γ2, and type IV collagen [204,205].

However, IHC alone cannot guarantee the stratification of OSCC patients that is needed for earlier diagnosis and targeted therapy. This is due to the inherent limitations of IHC, such as poor standardization reproducibility and difficulty in performing multiple assays [206].

MS has emerged as a promising platform to overcome many of these limitations. It is highly accurate and reproducible, and it can measure multiple analytes simultaneously to provide comprehensive proteomic information. 

The use of high-throughput proteomics in OSCC would allow:

− Understand the biology of this tumor.
− Individuate therapeutic targets and response markers.
− Identify predictive and prognostic biomarkers for clinical use.

In this regard, our review reports that proteomics found proteins associated with metabolic processes, including glycolysis, extracellular matrix remodeling, and hypoxia, to be overexpressed or downregulated in OSCC.

Thus, our review justifies a potential future clinical application of MS in OSCC. As for other tumor types, also for OSCC, proteomics could identify patients with a high or low probability of recurrence and/or metastasis, or patients responsive to or resistant to chemotherapy or radiotherapy.

In conclusion, this systematic review identified 28 proteins—namely ALDOA, CAD, CALR, CAV1, CSPG4, DEFA3, EFEMP1, ENPP2, FBLN2, FGA, FGG, HP1BP3, IGF2R, LAMC, LTBP2, LUM, MMP-1, 3, MMP-8, MMP-9, PKM, POSTN, STAT5A, RBM39, SF3B3, TGFBI, TNC, and VDAC1—which were observed as potentially capable of monitoring OSCC onset and/or progression.

In addition to providing information improving the understanding of oral carcinogenesis, these results encourage the combination of conventional tumor-focused treatments with therapeutic approaches modulating the activity of stromal cells.

## Figures and Tables

**Table 2:** Molecular functions retrieved from the UniProt database (https://www.uniprot.org (accessed on 27 January 2024)), variation in the expression, validation, and RNA expression of the 28 selected proteins. ns: not significant; NA: not applicable.

Protein Name	Protein ID	Molecular Function	Variation	Validation	RNA Expression
Fructose-bisphosphate aldolase A	ALDOA	Actin binding Cadherin binding Cytoskeletal protein binding Fructose binding Fructose-bisphosphate aldolase activity Identical protein binding RNA binding Tubulin binding	Up CAF [43] Down MSC [44]	/	ns
CAD protein	CAD	Aspartate binding Aspartate carbamoyltransferase activity ATP binding Carbamoyl-phosphate synthase (glutamine-hydrolyzing) activity Dihydroorotase activity Enzyme binding Identical protein binding Protein kinase activity Zinc ion binding”	Up tumor mass [36,37]	/	ns
Calreticulin	CALR	Calcium ion binding Carbohydrate binding Complement component C1q complex binding DNA binding Hormone binding Integrin binding Iron ion binding mRNA binding Nuclear androgen receptor binding Nuclear export signal receptor activity Peptide binding Protein-folding chaperone Protein-folding chaperone binding RNA binding Ubiquitin protein ligase binding Unfolded protein binding Zinc ion binding	Down CAF [38,41]	/	ns
Caveolin-1	CAV1	ATPase binding Cholesterol binding Enzyme binding Identical protein binding Inward rectifier potassium channel inhibitor activity Molecular adaptor activity Nitric oxide synthase binding Patched binding Peptidase activator activity Protein heterodimerization activity Protein kinase binding Protein sequestering activity Protein-containing complex binding Protein–macromolecule adaptor activity Signaling receptor binding small GTPase binding Transmembrane transporter binding	Up tumor mass [33,35]	IHC [33]	up
Chondroitin sulfate proteoglycan 4	CSPG4	Coreceptor activity Protein kinase binding	Down CAF [41] Up TIF [45]	/	ns
Neutrophil defensin 3	DEFA3	Protein homodimerization activity	Up tumor mass [30,34]	/	NA
EGF-containing fibulin-like extracellular matrix protein 1	EFEMP1	Calcium ion binding Epidermal growth factor receptor activity Epidermal growth factor receptor binding Growth factor activity	Down ECM [40] Up CAF [41]	/	ns
Ectonucleotide pyrophosphatase/ phosphodiesterase family member 2	ENPP2	Alkylglycerophosphoethanolamine phosphodiesterase activity Calcium ion binding Hydrolase activity Lysophospholipase activity Nucleic acid binding Phosphodiesterase I activity Polysaccharide binding Scavenger receptor activity Zinc ion binding	Down CAF [39,41]	/	ns
Fibulin-2	FBLN2	Calcium ion binding Extracellular matrix binding Extracellular matrix structural constituents Extracellular matrix constituents conferring elasticity	Down ECM [40] Down CAF [41]	/	ns
Fibrinogen alpha chain	FGA	Extracellular matrix structural constituents Metal ion binding Signaling receptor binding Structural molecule activity	Up tumor mass [30] Down ECM [40]	/	ns
Fibrinogen gamma chain	FGG	Cell adhesion molecule binding Extracellular matrix structural constituents Identical protein binding Metal ion binding Signaling receptor binding Structural molecule activity	Up tumor mass [30] Down ECM [40]	/	ns
Heterochromatin protein 1-binding protein 3	HP1BP3	DNA binding Nucleosome binding	Up tumor mass [36] Down tumor mass [37]	/	ns
Cation-independent mannose-6-phosphate receptor	IGF2R	Enzyme binding G-protein alpha-subunit binding Identical protein binding Insulin-like growth factor binding Insulin-like growth factor II binding Insulin-like growth factor receptor activity Mannose binding Phosphoprotein binding Retinoic acid binding Retromer complex binding Signaling receptor activity	Up tumor mass [36] Down MSC [44]	/	ns
Laminin subunit gamma-2	LAMC2	Cadherin binding Microtubule binding Microtubule plus end polymerase Microtubule plus-end binding Ribonucleoprotein complex binding	Up tumor mass [36] Up TIF [45]	/	up
Latent-transforming growth factor beta-binding protein 2	LTBP2	Calcium ion binding Growth factor binding Heparin binding Microfibril binding	Up tumor mass [36] Up CAF [39]	/	ns
Lumican	LUM	Collagen binding Extracellular matrix structural constituents confer compression resistance	Down CAF [38,41]	/	up
Interstitial collagenase	MMP-1	Endopeptidase activity Metalloendopeptidase Peptidase activity Serine-type endopeptidase activity zinc ion binding	Down CAF [38] Up CAF [42] Up MSC [44]	/	up
Stromelysin-1	MMP-3	Endopeptidase activity Metalloendopeptidase activity Metallopeptidase activity Peptidase activity Serine-type endopeptidase activity zinc ion binding	Up CAF [39] Down CAF [41]	qPCR and ELISA [39]	up
Neutrophil collagenase	MMP-8	Calcium ion binding Endopeptidase activity Metalloendopeptidase activity Metallopeptidase activity Peptidase activity Serine-type endopeptidase activity zinc ion binding	Up tumor mass [30] Up CAF [42] Up TIF [46]	IHC [46]	up
Matrix metalloproteinase 9	MMP-9	Collagen binding Endopeptidase activity Identical protein binding Metalloendopeptidase activity Metallopeptidase activity Peptidase activity Serine-type endopeptidase activity Zinc ion binding	Up tumor mass [30] Up CAF [42] Up TIF [46]	IHC [46]	up
Pyruvate kinase PKM	PKM	ATP binding Cadherin binding Histone H3T11 kinase activity Magnesium ion binding MHC class II protein complex binding mRNA binding Potassium ion binding Protein homodimerization activity Protein tyrosine kinase activity Pyruvate kinase activity RNA binding Transcription coactivator activity	Down tumor mass [30] Up CAF [43]	/	NA
Periostin	POSTN	Cell adhesion molecule binding Heparin binding Metal ion binding	Up tumor mass [33] Up TIF [45]	IHC [33]	up
RNA-binding protein 39	RBM39	RNA binding RS domain binding U1 snRNP binding	Up tumor mass [36] Up TIF [45]	/	ns
Splicing factor 3B subunit 3	SF3B3	Protein-containing complex binding U2 snRNA binding	Up tumor mass [36,37]	/	ns
Signal transducer and activator of transcription 5A	STAT5A	DNA-binding transcription factor activity DNA-binding transcription factor activity RNA polymerase II-specific DNA-binding transcription factor binding RNA polymerase II cis-regulatory region Sequence-specific DNA binding	Up tumor mass [30] Down epithelium [31]	/	ns
Transforming growth factor-beta-induced protein ig-h3	TGFBI	Cell adhesion molecule binding Collagen binding Extracellular matrix binding Extracellular matrix structural constituents Identical protein binding Integrin binding	Up CAF [39] Down MSC [44]	/	up
Tenascin	TNC	Extracellular matrix structural constituent Syndecan binding	Up tumor mass [33,36] Up TIF [45]	IHC [33]	up
Voltage-dependent anion-selective channel protein 1	VDAC1	Ceramide binding Cholesterol binding Identical protein binding Phosphatidylcholine binding Porin activity Protein kinase binding Transmembrane transporter binding Voltage-gated monoatomic anion channel activity	Up CAF [38] Down CAF [43]	/	ns

**Table 3:** GO enrichment analysis of significative biological processes associated with the 28 selected proteins.

#Term ID	Term Description	False Discovery Rate	Matching Proteins in Your Network
GO:0030198	Extracellular matrix organization	2.2 × 10^−6^	MMP-8, LUM, 3, MMP-1, CAV1, MMP-9, POSTN, FBLN2, TGFBI
GO:0030574	Collagen catabolic process	1.8 × 10^−3^	MMP-8, 3, MMP-1, MMP-9
GO:0022617	Extracellular matrix disassembly	2.4 × 10^−3^	MMP-8, 3, MMP-1, MMP-9
GO:0071492	Cellular response to UV-A	2.4 × 10^−3^	3, MMP-1, MMP-9
GO:0050789	Regulation of biological processes	1.5 × 10^−2^	MMP-8, RBM39, ENPP2, LTBP2, LAMC2, TNC, LUM, 3, SF3B3, CSPG4, PKM, CALR, MMP-1, DEFA3, FGG, CAV1, STAT5A, IGF2R, MMP-9, POSTN, EFEMP1, VDAC1, FBLN2, HP1BP3, TGFBI, ALDOA, FGA
GO:0016043	Cellular component organization	1.8 × 10^−2^	MMP-8, LTBP2, LAMC2, TNC, LUM, 3, SF3B3, CSPG4, CALR, MMP-1, FGG, CAV1, MMP-9, POSTN, FBLN2, HP1BP3, TGFBI, ALDOA, FGA
GO:0045907	Positive regulation of vasoconstriction	2.3 × 10^−2^	FGG, CAV1, FGA
GO:0048522	Positive regulation of the cellular process	2.3 × 10^−2^	MMP-8, ENPP2, LAMC2, TNC, LUM, 3, SF3B3, CSPG4, PKM, CALR, MMP-1, FGG, CAV1, STAT5A, IGF2R, MMP-9, VDAC1, FBLN2, FGA
GO:0150077	Regulation of the neuroinflammatory response	2.3 × 10^−2^	MMP-8, MMP-3, MMP-9
GO:0050794	Regulation of the cellular process	2.4 × 10^−2^	MMP-8, RBM39, ENPP2, LTBP2, LAMC2, TNC, LUM, 3, SF3B3, CSPG4, PKM, CALR, MMP-1, DEFA3, FGG, CAV1, STAT5A, IGF2R, MMP-9, POSTN, EFEMP1, VDAC1, FBLN2, HP1BP3, TGFBI, FGA
GO:0010811	Positive regulation of cell–substrate adhesion	3.4 × 10^−2^	CALR, FGG, FBLN2, FGA
GO:0032101	Regulation of response to external stimulus	3.7 × 10^−2^	MMP-8, TNC, 3, CALR, FGG, CAV1, MMP-9, FGA
GO:1900026	Positive regulation of substrate adhesion-dependent cell spreading	3.7 × 10^−2^	CALR, FGG, FGA
GO:0010634	Positive regulation of epithelial cell migration	5.0 × 10^−2^	ENPP2, CALR, STAT5A, MMP-9

**Table 4:** GO enrichment analysis of significative molecular functions associated with the 28 selected proteins.

#Term ID	Term Description	False Discovery Rate	Matching Proteins in Your Network
GO:0005201	Extracellular matrix structural constituents	1.6 × 10^−4^	TNC, LUM, FGG, FBLN2, TGFBI, FGA
GO:0005488	Binding	1.5 × 10^−2^	MMP-8, RBM39, ENPP2, LTBP2, LAMC2, CAD, TNC, LUM, 3, SF3B3, CSPG4, PKM, CALR, MMP-1, DEFA3, FGG, CAV1, STAT5A, IGF2R, MMP-9, POSTN, EFEMP1, VDAC1, FBLN2, HP1BP3, TGFBI, ALDOA, FGA
GO:0004222	Metalloendopeptidase activity	3.7 × 10^−2^	MMP-8, 3, MMP-1, MMP-9

**Table 5:** GO enrichment analysis of significative cellular components associated with the 28 selected proteins.

#Term ID	Term Description	False Discovery Rate	Matching Proteins in Your Network
GO:0031012	Extracellular matrix	1.7 × 10^−16^	MMP-8, LTBP2, LAMC2, TNC, LUM, 3, CSPG4, PKM, CALR, MMP-1, FGG, MMP-9, POSTN, EFEMP1, FBLN2, TGFBI, FGA
GO:0062023	Collagen-containing extracellular matrix	1.4 × 10^−15^	MMP-8, LTBP2, LAMC2, TNC, LUM, CSPG4, PKM, CALR, FGG, MMP-9, POSTN, EFEMP1, FBLN2, TGFBI, FGA
GO:0005576	Extracellular region	5.4 × 10^−9^	MMP-8, ENPP2, LTBP2, LAMC2, CAD, TNC, LUM, 3, CSPG4, PKM, CALR, MMP-1, DEFA3, FGG, IGF2R, MMP-9, POSTN, EFEMP1, VDAC1, FBLN2, TGFBI, ALDOA, FGA
GO:0005615	Extracellular space	5.6 × 10^−9^	MMP-8, ENPP2, LTBP2, LAMC2, CAD, TNC, LUM, 3, CSPG4, PKM, CALR, DEFA3, FGG, IGF2R, MMP-9, POSTN, EFEMP1, VDAC1, TGFBI, ALDOA, FGA
GO:1903561	Extracellular vesicle	9.8 × 10^−7^	LTBP2, CAD, LUM, CSPG4, PKM, CALR, DEFA3, FGG, IGF2R, MMP-9, EFEMP1, VDAC1, FBLN2, TGFBI, ALDOA, FGA
GO:0070062	Extracellular exosome	5.8 × 10^−6^	LTBP2, CAD, LUM, CSPG4, PKM, CALR, DEFA3, FGG, IGF2R, MMP-9, EFEMP1, VDAC1, TGFBI, ALDOA, FGA
GO:0030141	Secretory granule	3.9 × 10^−5^	MMP-8, PKM, CALR, DEFA3, FGG, CAV1, IGF2R, MMP-9, ALDOA, FGA
GO:0031982	Vesicle	8.7 × 10^−5^	MMP-8, LTBP2, CAD, LUM, CSPG4, PKM, CALR, DEFA3, FGG, CAV1, IGF2R, MMP-9, EFEMP1, VDAC1, FBLN2, TGFBI, ALDOA, FGA
GO:0071944	Cell periphery	2.6 × 10^−4^	MMP-8, ENPP2, LTBP2, LAMC2, TNC, LUM, 3, CSPG4, PKM, CALR, MMP-1, FGG, CAV1, IGF2R, MMP-9, POSTN, EFEMP1, VDAC1, FBLN2, TGFBI, FGA
GO:0034774	Secretory granule lumen	8.0 × 10^−4^	MMP-8, PKM, DEFA3, FGG, ALDOA, FGA
GO:1904724	Tertiary granule lumen	9.0 × 10^−3^	MMP-8, MMP-9, ALDOA
GO:0005577	Fibrinogen complex	9.9 × 10^−3^	FGG, FGA
GO:0070013	Intracellular organelle lumen	1.1 × 10^−2^	MMP-8, RBM39, CAD, TNC, LUM, SF3B3, CSPG4, PKM, CALR, DEFA3, FGG, STAT5A, IGF2R, MMP-9, VDAC1, HP1BP3, ALDOA, FGA
GO:0031093	Platelet alpha granule lumen	1.2 × 10^−2^	FGG, ALDOA, FGA
GO:0005925	Focal adhesion	2.5 × 10^−2^	TNC, CSPG4, CALR, CAV1, IGF2R
GO:0005604	Basement membrane	3.1 × 10^−2^	LAMC2, TNC, TGFBI
GO:0005796	Golgi lumen	3.7 × 10^−2^	LUM, CSPG4, DEFA3

## Supplementary Materials

The following supporting information can be downloaded at: https://www.mdpi.com/article/10.3390/ijms25168929/s1.
