# Supplementary material for: Biomarkers Identification in the Microenvironment of Oral Squamous Cell Carcinoma: A Systematic Review of Proteomic Studies
Source: Int J Mol Sci. 2024 Aug 16;25(16):8929. doi: 10.3390/ijms25168929 (PMC11354375; doi:10.3390/ijms25168929)
Supplement: Supplementary file 1 [file ijms-25-08929-s001.zip › ijms-3104788-sl.pdf]

## Supplementary Figure 1

**a**

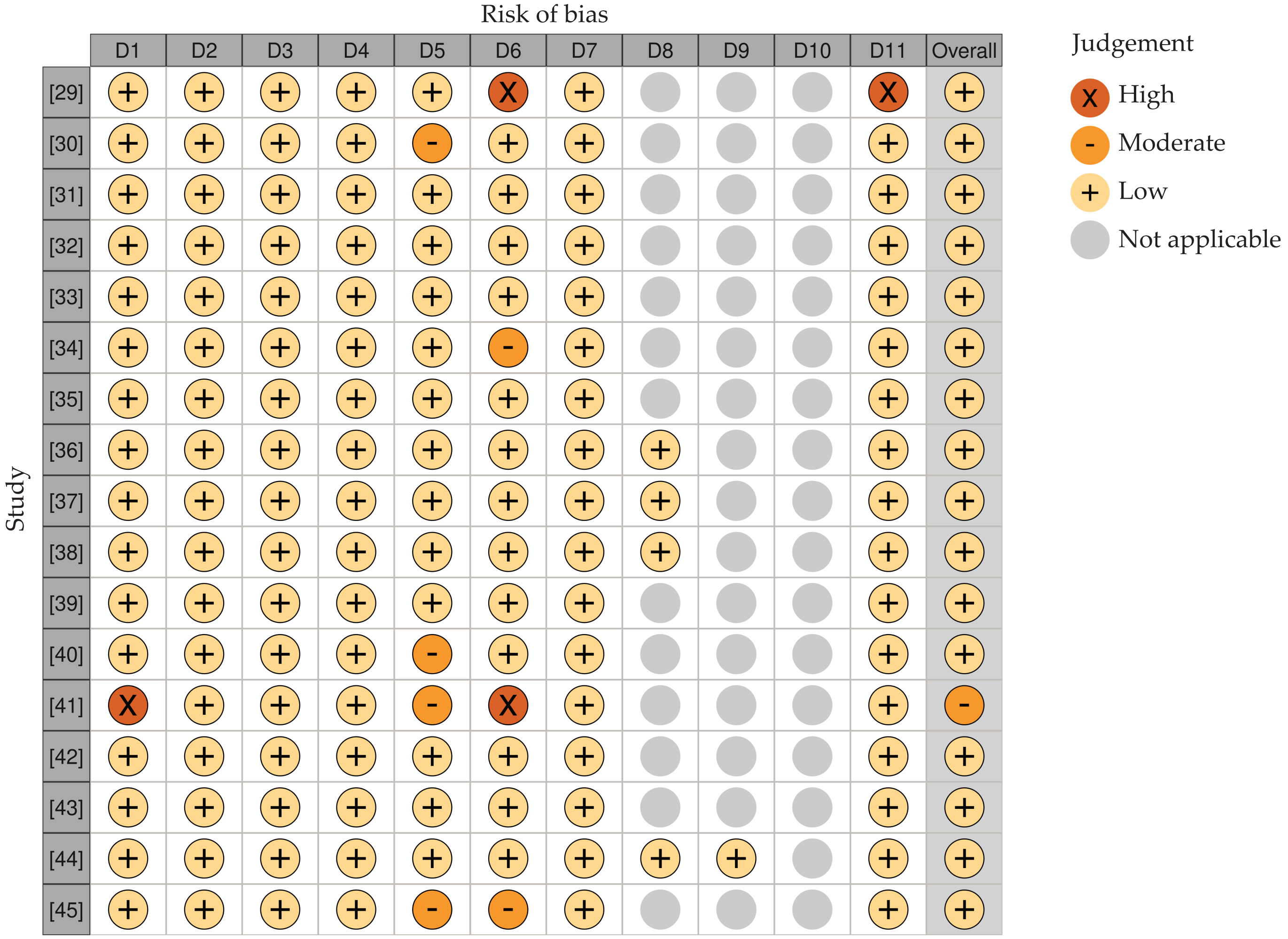


D1: Were the two groups similar and recruited from the same population?
D2: Were the exposures measured similarly to assign people to both exposed and unexposed groups?
D3: Was the exposure measured in a valid and reliable way?
D4: Were confounding factors identified?
D5: Were strategies to deal the confounding factors stated?
D6: Were the groups/participants free of the outcome at the start of the study (or at the moment of exposure)?
D7: Were the outcomes measured in a valid and reliable way?
D8: Was the follow up time reported and sufficient to be long enough for outcomes to occur?
D9: Was follow up complete, and if not, were the reasons to loss to follow up described and explored?
D10: Were strategies to address incomplete follow up utilized?
D11: Was appropriate statistical analysis used?

**b**

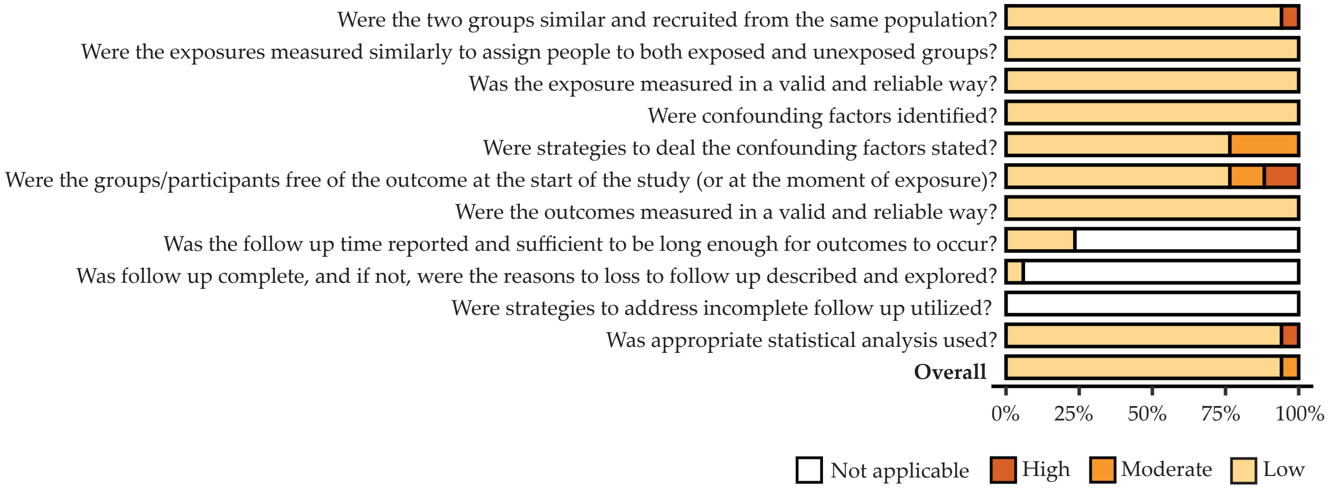


**Supplementary Figure 1.** (**a**) Traffic light plot of the risk of bias according to The Joanna Briggs Institute Critical Appraisal tool for each of the selected studies. (**b**) Bar blot depicting the percentage of the bias risk according to The Joanna Briggs Institute Critical Appraisal tool.

Supplementary Figure 2

**a**

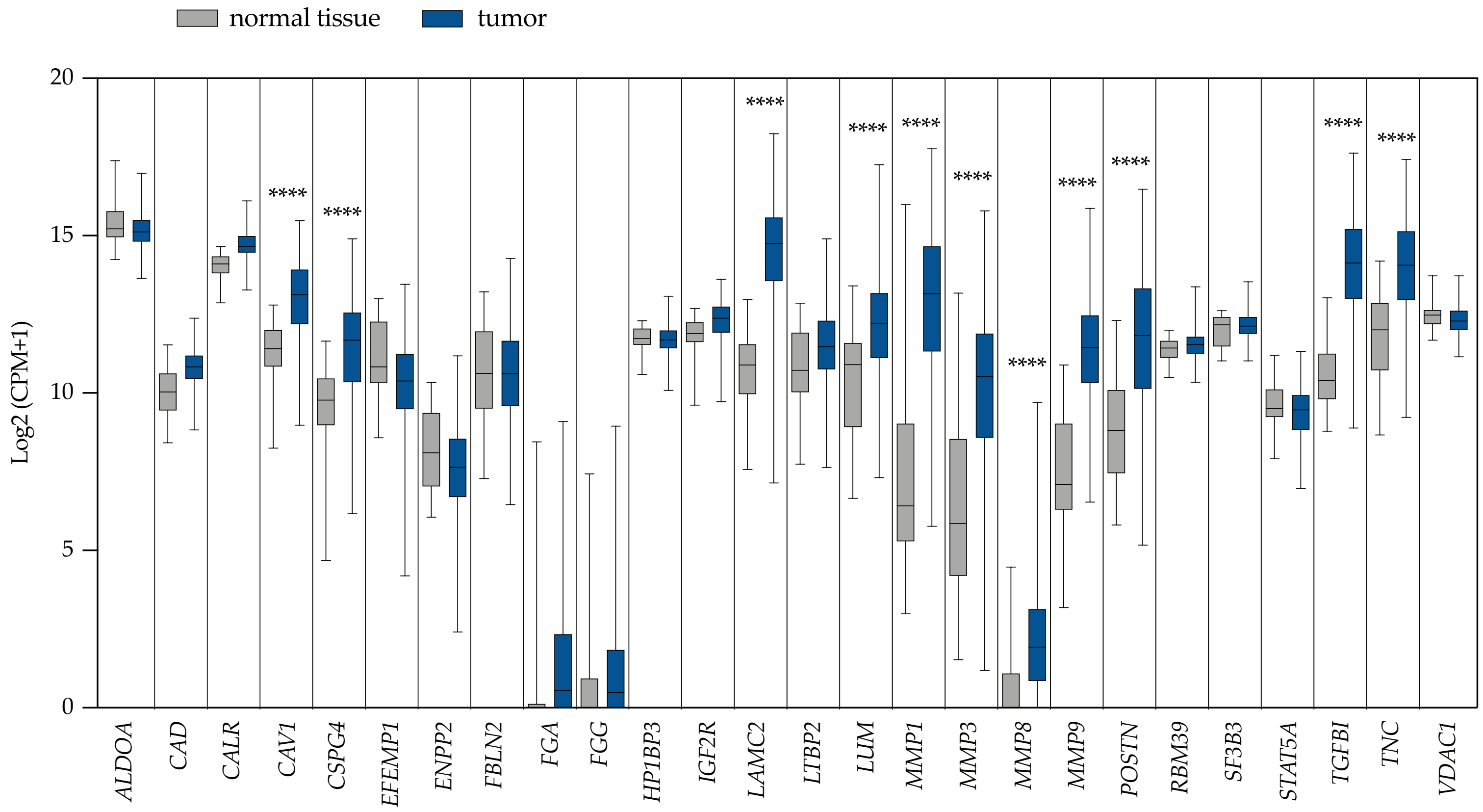


**Supplementary Figure 2.** Box plots depicting RNA-seq analysis of identified protein-coding gene expressions in 321 OSCC tumors from the TCGA Pan Cancer Atlas, and matched normal oral tissues. One-way ANOVA for multiple comparisons. **** $p < 0.0001$. CPM; counts per million mapped reads.
